# Association between Big Five Personality Traits and Participation in Cardiac Rehabilitation in Japanese Patients with Cardiovascular Disease: A Retrospective Cohort Study

**DOI:** 10.3390/ijerph18168589

**Published:** 2021-08-14

**Authors:** Takuji Adachi, Yuki Tsunekawa, Akihito Matsuoka, Daisuke Tanimura

**Affiliations:** 1 Department of Integrated Health Sciences, Nagoya University Graduate School of Medicine, Nagoya 461-8673, Japan; 2 Department of Rehabilitation, Nagoya Ekisaikai Hospital, Nagoya 454-8502, Japan; road_of_freedom_in2008@yahoo.co.jp (Y.T.); yaammatsuoka@yahoo.co.jp (A.M.); 3 Department of Cardiology, Nagoya Ekisaikai Hospital, Nagoya 454-8502, Japan; taniyan6dec@yahoo.co.jp

**Keywords:** personality traits, cardiac rehabilitation, cardiovascular disease, health-related behaviour

## Abstract

Cardiac rehabilitation (CR) remains underutilised, despite its established clinical benefit. A personality traits assessment may help promote CR implementation, as they are determinants of health-related behaviour. This study aimed to examine the association between the Big Five personality traits and outpatient CR participation in patients with cardiovascular disease (CVD) after discharge. This retrospective cohort study included 163 patients aged <80 years, who underwent inpatient CR when hospitalised for CVD. The Big Five personality traits (conscientiousness, neuroticism, openness, extraversion, and agreeableness) of each patient were evaluated at discharge, using the Japanese version of the Ten-Item Personality Inventory. We examined the relationship of each personality trait with non-participation in outpatient CR and dropout within three months, using logistic regression analysis. Out of 61 patients who initiated the outpatient CR, 29 patients dropped out, leaving us with 32 subjects. The logistic regression analysis results showed that high conscientiousness was associated with non-participation in CR. The primary reason for this was a lack of motivation. Conversely, low conscientiousness and high openness were predictors of dropout. This study suggests that the assessment of the Big Five personality traits, especially conscientiousness and openness, can help improve health communication with patients to promote outpatient CR participation after discharge.

## 1. Introduction

Cardiovascular disease (CVD) is a significant health concern worldwide [1]. In particular, secondary prevention has become more important in clinical practice because survival rates of CVD have improved with the advance of medical treatment and the emergency medical system [2]. Cardiac rehabilitation (CR) is a disease management programme that improves exercise tolerance [3,4], health-related quality of life [5,6], and prognosis [7,8]. However, the implementation rate of outpatient CR in Japan has been low compared to Western countries [9,10]. Additionally, previous studies have reported the dropout rate of CR within 3–6 months as 30–60% [11], indicating the evidence–practice gap in secondary prevention. A recent systematic review has demonstrated that many interpersonal factors are associated with non-participation in CR [12]. This evidence suggests the importance of patient–medical staff communication for promoting CR participation.

Personality traits are predictors of health-related behaviour over the life span, and may help in understanding patients’ interests or concerns in CR. The Big Five model, also known as the five-factor model, is a widely accepted model of personality traits [13,14], and has a close link to long-term health outcomes [15]. As the name suggests, it includes five personality traits: conscientiousness, neuroticism, openness, extraversion, and agreeableness. In brief, conscientiousness reflects the propensity to be self-controlled, task- and goal-directed, planful, and rule-following. Neuroticism contrasts even-temperedness with the experience of anxiety, worry, anger, and depression. Openness refers to the proneness to be original, complex, creative, and open to new ideas. Extraversion refers to the propensity to be sociable, active, assertive, and to experience positive affect. Finally, agreeableness refers to the degree to which a person needs pleasant and harmonious relationships with others [13,14].

Previous reports have demonstrated the strong relationships between lower conscientiousness and higher neuroticism with several adverse health-related behaviours, such as physical inactivity, smoking, alcohol consumption, and unhealthy eating [16,17,18]. Additionally, conscientiousness and neuroticism have been associated with adherence to medication and doctor’s regimens [19]. These results suggest that personality traits may become a clue for effective communication to promote patient engagement in CR.

To date, few studies have explored the relationship between the Big Five personality traits and CR participation. Therefore, the aim of this study was to examine the association between Big Five personality traits and outpatient CR participation in patients with CVD.

## 2. Materials and Methods

### 2.1. Study Design

The study design was a retrospective cohort study.

### 2.2. Subjects

We retrospectively retrieved data for patients with CVD admitted to Nagoya Ekisaikai Hospital in Nagoya City, Japan, between November 2019 and January 2021. Inclusion criteria were as follows: aged <80 years old, participated in inpatient CR programme, and answered a questionnaire during hospitalisation as routine clinical assessment. Patients with one or more of the following reasons were excluded: unable to answer the questionnaire due to visual or hearing impairment, severe psychiatric or neurological disorders, physician diagnosed dementia or anti-dementia drugs (N06D of Anatomical Therapeutic Chemical Classification System) before admission, and severe cognitive decline defined as ≤4 points on the Rapid Dementia Screening Test [20]. We excluded patients aged ≥80 years because of the increased prevalence of the need of assistance for outdoor walking, resulting in non-participation in CR regardless of the patient’s will. An opt-out method for participant recruitment was used for this retrospective study, and written informed consent was not required from those included in the study.

### 2.3. Personality Traits

Personality traits for each patient were measured using the Japanese version of the Ten-Item Personality Inventory (TIPI-J) in routine clinical practice [21]. To date, the original TIPI has been widely used as the brief scale for the Big Five evaluation [22]. The results of previous studies suggested the importance of assessing multiple aspects of personality traits for predicting health behaviour [16,17,18,19]. We used the TIPI for patient assessment because of its simplicity in measuring multiple personality traits in clinical practice.

The reliability and validity of the TIPI-J have been reported [21]. The TIPI-J includes two items assessing each of the Big Five domains, comprising ten items. Each domain is assessed by one item positively keyed and the other negatively keyed. Each item is answered using a 7-category response scale. The averages of the two-item scores included in each trait were calculated to provide each trait score (ranging from 1–7), with higher scores indicating a higher trait level. Reliability and concurrent validity of TIPI-J have also been confirmed among older individuals [23].

### 2.4. Study Outcome: Participation in Outpatient Cardiac Rehabilitation

In Japan, CR has been fully covered by health insurance since 2007. The Nagoya Ekisaikai Hospital provides in- and outpatient CR, according to the guidelines of the Japanese Circulation Society [24], which recommends that CR begins with supervised CR followed by a combination of supervised and non-supervised CR 3–5 times/wk. Based on the standard CR programme recommended by the guidelines mentioned above, CR included exercise training, patient education, and patient counselling. Exercise training comprised preparatory exercises, aerobic exercises, and cool-down. Exercise intensity was set at each patient’s anaerobic threshold, measured using cardiopulmonary exercise testing, the Karvonen formula, or an intensity of 11–13 on the Borg scale. A nationwide survey in Japan has reported the safety of exercise-based CR [25]. Furthermore, resistance training was generally added to the aerobic exercise. Functional training, such as individually tailored physical therapy and balance training was provided for physically frail patients or patients with physical impairment to improve performance of activities in daily living [24].

Each patient was asked to complete a self-administered questionnaire related to outpatient CR, as simultaneously completing TIPI-J. The questionnaire included the will to participate in outpatient CR after discharge (yes or no) and the reasons for non-participation in CR. Non-participation reasons were asked among those who did not plan to participate in outpatient CR. The reason for each patient was chosen from the following: lack of transportation, return to work, declined physical strength (subjective physical weakness or utilisation of long-term care), lack of motivation for CR, economic reason, and others.

From medical records, we examined the outpatient CR attendance for each patient for three months after discharge, which has been associated with a favourable prognosis in patients with CVD [26]. To stratify patients into those who continued outpatient CR for three months and those who dropped out, we defined the participation group as attending at least one CR session a month within three months after discharge. Furthermore, the dropout group was defined as those who did not continue CR after being discharged, or continued but for less than three months after being discharged. Thus, patients were stratified into non-participation, participation, and dropout groups, and these CR participation patterns were considered as the study outcome.

In Japan, the first coronavirus disease 2019 (COVID-19) case was reported on 16 January 2020. During the state of emergency by the government on 7 April (until 6 May), one-third of the training facilities for cardiac rehabilitation continued to provide an outpatient programme [27]. We prohibited patients with common cold symptoms, such as fever, from participating in CR, and all patients wore masks in the exercise room. We conducted careful outpatient CR, disinfected the ergometer handles and saddles with alcohol before and after each procedure, and completely separated outpatients from inpatients. These prophylactic procedures were conducted according to the guidelines by the European Association of Preventive Cardiology [28].

### 2.5. Assessment of Depression

Depression was screened using the Hospital Anxiety and Depression Scale (HADS) [29] simultaneously with TIPI-J. The HADS is a widely used questionnaire for depression and anxiety in population studies, primary care, and hospital settings [30]. It consists of two 7-item self-reported subscales designed to assess current depressive and anxiety symptomatology, respectively. Each item is scored from 0 (not present) to 3 (maximally present), and scores on both the HADS sub-scores for anxiety (HADS-anxiety) and depression (HADS-depression) range from 0 to 21. In this study, only the depression score was calculated for each patient, since anxiety was not measured for all patients as a routine assessment.

### 2.6. Patient Characteristics

The demographic measures and clinical data were collected from medical records as patient characteristics. Clinical data included aetiology, comorbidities, left ventricular ejection fraction, biochemical data, medications, depression, and walking ability. Walking ability was assessed by the need for a walking device or assistance.

### 2.7. Sample Size Calculation

Currently, there are no reports assessing TIPI in patients with CVD, and there are no prior studies to help calculate the sample size. Additionally, this study was a retrospective study using medical records, and did not prospectively enrol patients based on precise sample size calculation. However, we retrospectively retrieved data to allow adjustment for age and gender in a multivariate logistic regression analysis, with CR participation patterns after discharge as the outcome variable. Thus, according to the concept of one variable per 10 outcome events [31], we reviewed medical records to include at least around 30 patients for each of the non-participation, participation, and dropout groups.

### 2.8. Statistical Analysis

Patients with missing data for TIPI-J and CR participation were excluded from all the analyses. Continuous variables were expressed as mean and standard deviation for normally distributed variables, and as median with interquartile range for non-normally distributed data. Categorical data were expressed as numbers and percentages.

Analysis of variance with post hoc Tukey testing or Kruskal–Wallis method with post hoc Steel–Dwass testing was performed to compare continuous variables across three groups (i.e., non-participation, participation, dropout) as appropriate. Categorical variables were compared by the chi-square test. The Bonferroni correction was applied for multiple tests to reduce the probability of making type I errors.

In assessing the association between personality traits and outpatient CR, binary logistic regression analysis was performed with non-participation or dropout as a dependent variable and each personality trait as an independent variable. Interactions were not considered in this study, as we aimed to explore the association between each personality trait and non-participation or dropout. Due to the limited sample size, we adjusted only for age and gender, according to the concept of one variable per 10 outcome events in the logistic regression model [31].

The primary reasons for non-participation in CR reported at discharge were aggregated according to the median of each personality trait. The prevalence of each reason was compared with the level of each personality trait using the chi-square test. All statistical analyses were conducted with Stata 15 (Stata Corporation, TX, USA) and R version 3.1.2 (R Foundation for Statistical Computing, Vienna, Austria). A p-value of <0.05 was considered statistically significant.

## 3. Results

**Figure 1:**
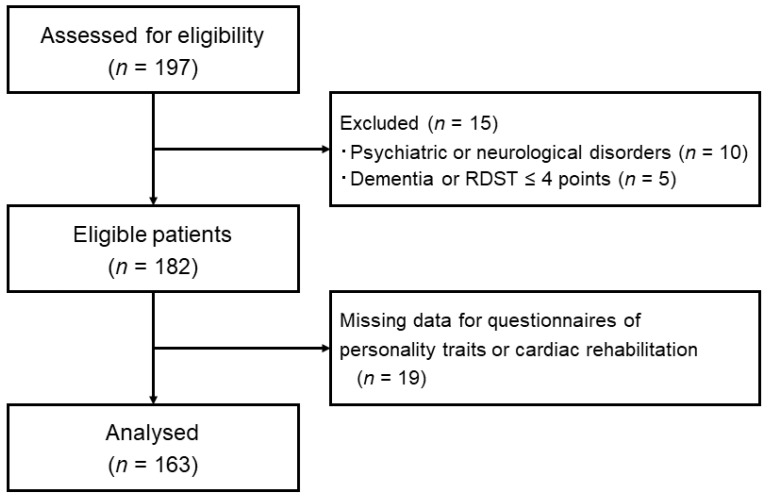
Flowchart of patient selection. RDST, Rapid Dementia Screening Test.

Of the 197 patients, 15 were excluded from the study based on the exclusion criteria. A total of 163 patients were included in the present analysis, after excluding 19 patients with missing data for questionnaires of personality traits or CR participation (Figure 1). The characteristics of the study participants are presented in Table 1. The mean age was 69.2 ± 10.6 years, and 72.4% of the participants were men. The prevalence of acute coronary syndrome (ACS), heart failure, and cardiac surgery as a principal diagnosis for hospitalisation were 32.5%, 31.9%, and 30.7%, respectively. Patients excluded due to missing data (*n* = 19) had similar characteristics to those included in the analysis (age: 70.1 ± 11.7 years, men: 68.4%, ACS: 36.8%, heart failure: 31.6%, cardiac surgery: 26.3%). The other characteristics also showed no statistically significant differences, although the prevalence of diabetes tended to be higher in patients included than in those excluded due to missing data (38% vs. 15.8%, *p* = 0.06).

Of the 61 patients who initiated outpatient CR after discharge, 32 patients participated in at least one CR session a month during the three-month follow-up (participation group), whereas 29 patients did not (dropout). The dropout group was younger than the non-participation group (65.2 ± 10.0 vs. 70.8 ± 11.0 years, *p* < 0.05). There were no statistically significant differences in the other characteristics among the three groups (*p* > 0.05).

**Figure 2:**
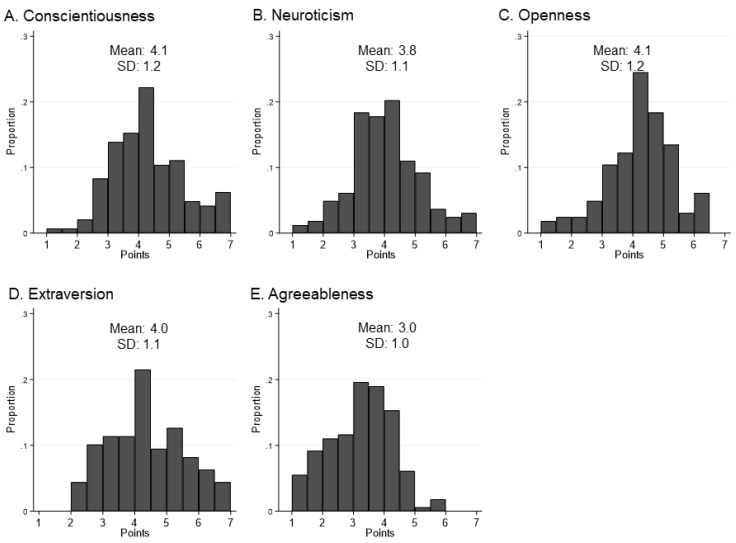
Distribution of each personality trait in overall patients (*n* = 163). All personality traits are normally distributed (*p* for Shapiro–Wilk test > 0.05). SD, standard deviation.

The Big Five personality traits were normally distributed (*p* for Shapiro–Wilk test > 0.05), as shown in Figure 2. Table 2 shows the Big Five personality traits according to CR participation. Conscientiousness was significantly different across the groups (*p* < 0.001), and the post hoc test showed that there was a significant difference between the non-participation group and the dropout group (4.4 ± 1.3 vs. 3.4 ± 0.8, *p* < 0.05). Furthermore, there was a substantial difference in openness among the groups (*p* < 0.001), and the dropout group showed a higher openness level (4.6 ± 0.9), compared to the participation group (3.6 ± 0.9, *p* < 0.05) and the non-participation group (3.9 ± 1.1, *p* < 0.05). The other three personality traits did not show statistical differences across the groups.

**Figure 3:**
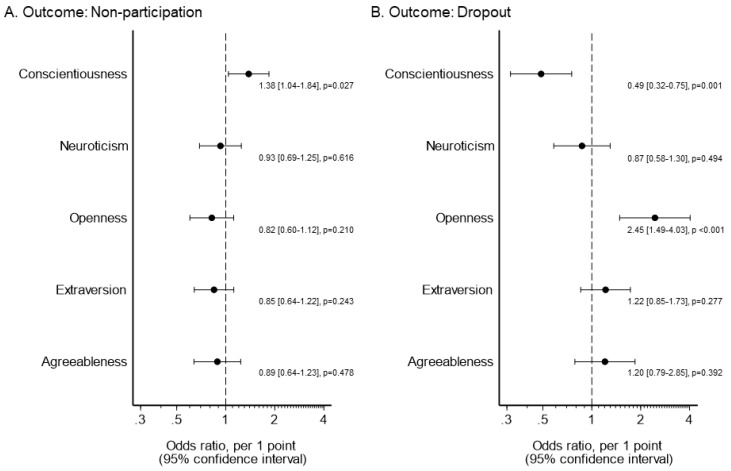
Results of logistic regression analysis (*n* = 163). (**A**) Dependent variable: non-participation in outpatient cardiac rehabilitation. (**B**) Dependent variable: dropout from outpatient cardiac rehabilitation. Adjusted for age and gender.

The results of the logistic regression analysis adjusted for age and gender are shown in Figure 3. Higher conscientiousness was associated with non-participation (odds ratio 1.38, per 1 point; 95% confidence interval 1.04–1.84). Alternatively, lower conscientiousness (odds ratio 0.49, per 1 point; 0.32–0.75) and higher openness (odds ratio 2.45, per 1 point; 1.49–4.03) were associated with dropouts.

**Figure 4:**
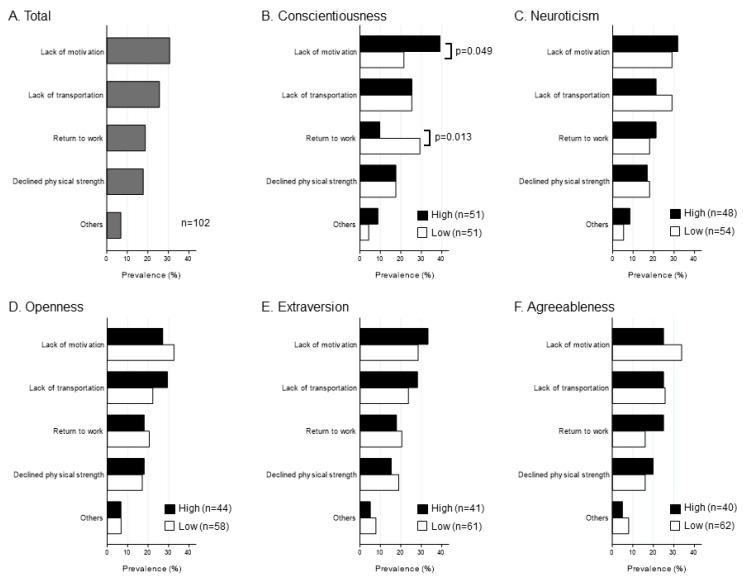
Reasons for non-participation in outpatient CR according to the median level of each personality trait (*n* = 102). *p* < 0.05 for chi-square test (high level vs. low level). CR, cardiac rehabilitation.

The primary reasons for non-participation in CR (*n* = 102) are presented in Figure 4. Lack of motivation was the most common reason for non-participation. No patient mentioned economic conditions or COVID-19 as the reason for non-participation in CR. Furthermore, the prevalence of lack of motivation was high in a high conscientiousness group. When stratified by the other traits, there were no apparent differences in the primary reasons for non-participation in CR.

## 4. Discussion

This study explored the potential association between the Big Five personality traits and outpatient CR participation. Consequently, high conscientiousness was associated with non-participation in CR, whereas low conscientiousness and high openness were predictors of dropout from CR. To our knowledge, this is the first study to examine the association between CR participation and the Big Five personality traits, which have been reported to be associated with health-related behaviours in the field of public health.

CR has remained underutilised, despite the established benefits for secondary prevention. A recently updated systematic review suggested that face-to-face recruitment by healthcare providers improves patient utilisation of CR [32]. These results are consistent with recent guidelines that have recommended shared decision making to enhance patient-centred care [33]. Considering the patient priority assessment as the first step of shared decision making [34], an understanding of the patient’s personality traits could help patient–medical staff communication. This study’s strength is that it investigated the clinical significance of the Big Five personality traits for secondary CVD prevention. Previous reports had only studied the Big Five in the field of primary prevention for the general population. Nevertheless, this study should be considered as hypothesis generating, due to its small sample size; our results will serve as preliminary data for considering the health communication with patients, in order to promote CR.

The results of this study suggest that lower conscientiousness predicts dropout from CR within three months. This result was consistent with previous studies that reported high conscientiousness as an associated factor of favourable health-related behaviours, including an active lifestyle, healthy eating, and adherence to medication and doctor’s regimens [16,17,18]. Contrary to our expectation, a high conscientiousness level was associated with non-CR participation. Our result could be partly explained by the association between conscientiousness and self-efficacy for disease management. A previous study in CVD patients demonstrated that high conscientiousness was associated with high self-efficacy for cardiovascular treatment [35], a known associated factor of non-adherence to CR [12]. The present study supports another recent study reporting that high health literacy was associated with dropout from CR [36]. A high prevalence of lack of motivation for outpatient CR among the high conscientiousness group seems to support our interpretation. The above implies that it might be imperative for staff to explain and promote the benefits of the CR programme to those patients with high consciousness, as this may be important for overcoming barriers to CR [37].

In addition, subgroup analyses based on gender and social factors may help in interpreting this unexpected result. For example, women generally have lower cardiovascular risk than men, due to the favourable influence of oestrogen [38], but lower lifestyle adherence, due to the influence of social roles [39,40]. This has been recognised as a lifestyle paradox in women. Therefore, further detailed analyses are required to clarify the reasons of the negative correlation between conscientiousness and CR participation.

Another finding of this study was the association between high openness and CR dropout. A recent study demonstrated that high openness was associated with preventive healthcare utilisation, such as mammograms and breast lump checks in women [41]. The correlation between openness and a physically active lifestyle has also been reported [16]. Our results were not consistent with these previous results showing the potentially favourable effects of openness on health-related behaviour in the field of public health. However, several studies suggested that this personality trait did not necessarily positively affect adherence to medical treatment. For instance, openness has been linked to complementary and alternative medicine, and it could be that this personality trait is associated with acceptance towards more unconventional forms of medicine [42]. From our results, patients with high openness are likely to be prone to a decline in adherence or incompletion of the CR programme. In such cases, medical professionals may need to frequently provide health information or feedback regarding their lifestyle to maintain their interest in CR.

Although this study provided the hypotheses on the association between personality traits and CR participation, further studies are needed for improving patients’ adherence to medical intervention. Previous intervention studies of exercise-based CR showed that only approximately 40% of patients reported being compliant with exercise recommendations [37]. The present study divided patients into three groups based on outpatient CR participation, and there was a lack of data on patients’ adherence after discharge. According to the European Society of Cardiology position statement, patients’ adherence can be classified into three levels based on compliance to the prescribed therapeutic regimen [37]. This statement has also documented many inter- and intrapersonal factors as barriers to exercise and non-participation in CR. Given the above, prospective studies that analyse the Big Five personality traits and follow-up data on health-related behaviours related to secondary prevention after discharge are needed.

This study has several limitations. First, there was potential for selection bias in the present analysis because of the single centre retrospective study. Hence, the generalisability of our results should be carefully discussed. Second, because of the limited sample size, confounding factors were not fully considered in the multivariate analysis. Subgroup analysis stratified by diagnosis may also be needed because of the potential differences in patient characteristics. Third, there may be unknown confounding factors, such as educational level and physical activity, that were not assessed in this study. Fourth, as mentioned before, we did not assess behavioural change or adherence to medical intervention, including medications and exercise after discharge. The influence of personality traits on clinical outcomes of outpatient CR (e.g., clinical events, exercise capacity) will also be next topics. These issues need to be addressed by further studies with a larger sample size and follow-up data. Finally, our findings may be affected by the COVID-19 pandemic, although it did not become a primary reason for non-participation in CR. Nevertheless, the present study has clinical significance in showing the potential usefulness of assessing personality traits for promoting CR participation.

## 5. Conclusions

Our results suggest that assessing the Big Five personality traits, particularly conscientious and openness, may assist in the health communication with patients to promote outpatient CR participation after discharge. Large-scale studies that include enough potential confounders and follow-up data on patients’ adherence are needed to confirm the hypothesis generated in this study.

## Figures and Tables

**Table 1:** Characteristics of the study participants.

	Overall (*n* = 163)	Non-Participation (*n* = 102)	Participation (*n* = 32)	Dropout (*n* = 29)	*p*
Age, years	69.2 ± 10.6	70.8 ± 11.0	67.8 ± 8.8	65.2 ± 10.0 ^§^	0.029 *
Male, n (%)	118 (72.4)	71 (69.6)	24 (75.0)	23 (79.3)	0.55 ^†^
Body mass index, kg/m^2^	21.8 (20.7–24.7)	21.5 (20.6–23.0)	22.0 (20.9–22.1)	25.7 (20.6–26.6)	0.48 ^‡^
Reason for hospitalisation, n (%)					0.18 ^†^
Acute coronary syndrome	53 (32.5)	31 (30.4)	14 (43.8)	8 (27.6)	
Heart failure	52 (31.9)	36 (35.3)	5 (15.6)	11 (37.9)	
Cardiac surgery	50 (30.7)	28 (27.4)	13 (40.6)	9 (31.0)	
Others	8 (4.9)	7 (6.9)	0 (0)	1 (3.5)	
Comorbidities, n (%)					
Hypertension	124 (76.1)	75 (73.5)	27 (84.4)	22 (75.9)	0.45 ^†^
Diabetes mellitus	62 (38.0)	40 (39.2)	12 (37.5)	10 (34.5)	0.90 ^†^
Dyslipidemia	77 (47.2)	42 (41.2)	19 (59.4)	16 (55.2)	0.13 ^†^
Prior heart failure	36 (22.1)	22 (21.6)	9 (28.1)	5 (17.2)	0.58 ^†^
Stroke	22 (14.7)	17 (16.7)	4 (12.5)	1 (3.4)	0.18 ^†^
COPD	10 (6.1)	5 (4.9)	4 (12.5)	1 (3.4)	0.24 ^†^
Cancer	15 (9.2)	10 (9.8)	2 (6.3)	3 (10.3)	0.81 ^†^
Left ventricular ejection fraction, %	51 (48–59)	52 (44–60)	48 (35–57)	50 (45–59)	0.34 ^‡^
Medications, n (%)					
Beta blocker	126 (77.3)	75 (73.5)	27 (84.4)	24 (82.8)	0.33 ^†^
ACEi/ARB	91 (55.8)	52 (51.0)	18 (56.3)	21 (72.4)	0.12 ^†^
MRA	56 (34.4)	31 (30.4)	16 (50.0)	9 (31.0)	0.12 ^†^
Diuretic	70 (42.9)	42 (41.2)	18 (56.3)	10 (34.5)	0.19 ^†^
Statin	96 (58.9)	55 (53.9)	20 (62.5)	21 (72.4)	0.18 ^†^
Anticoagulant	72 (44.2)	43 (42.2)	15 (46.9)	14 (48.3)	0.79 ^†^
Antithrombotic agent	108 (66.3)	67 (65.7)	20 (62.5)	21 (72.4)	0.70 ^†^
Depression score, points	8.8 ± 2.8	9.0 ± 2.6	8.8 ± 3.0	8.0 ± 3.1	0.29 *
Living alone, n (%)	41 (25.3)	21 (20.8)	9 (28.1)	11 (37.9)	0.16
Walking device or assistance, n (%)					0.16 ^†^
None	132 (80.9)	77 (75.5)	29 (90.6)	26 (89.7)	
Walking device	25 (15.4)	19 (18.6)	3 (9.4)	3 (10.3)	
Assistance	6 (3.7)	6 (5.9)	0 (0)	0 (0)	

Continuous variables are presented by mean ± standard deviation or median (interquartile range). Categorical variables are expressed by percentage. * *p* for analysis of variance. ^†^ p for chi-square test. ^‡^ *p* for Kruskal–Wallis test. ^§^ *p* < 0.05 vs. non-participation (after Bonferroni correction). COPD, chronic obstructive pulmonary disease; ACEi, angiotensin converting enzyme inhibitor; angiotensin II receptor blocker; MRA, mineralocorticoid receptor blocker.

**Table 2:** Comparisons of personality traits at discharge among groups based on the participation in outpatient CR for three months.

	Overall (*n* = 163)	Non-Participation (*n* = 102)	Participation (*n* = 32)	Dropout (*n* = 29)	*p* *
Conscientiousness	4.1 ± 1.2	4.4 ± 1.3	4.2 ± 1.3	3.4 ± 0.8 ^†^	<0.001
Neuroticism	3.8 ± 1.1	3.7 ± 1.1	4.0 ± 1.3	3.8 ± 1.1	0.364
Openness	4.1 ± 1.2	3.9 ± 1.1	3.6 ± 0.9	4.6 ± 0.9 ^†,‡^	<0.001
Agreeableness	3.0 ± 1.0	2.9 ± 1.0	3.1 ± 1.3	3.2 ± 0.9	0.281
Extraversion	4.0 ± 1.1	4.1 ± 1.2	4.3 ± 1.2	4.5 ± 1.3	0.218

* *p* for analysis of variance. ^†^ *p* < 0.05 for vs. non-participation group (after Bonferroni correction). ^‡^ *p* < 0.05 for vs. participation group (after Bonferroni correction). CR, cardiac rehabilitation.

## Data Availability

All data generated or analysed during this study are included in this published article.
